# Emergency undocking in robotic surgery: an international Delphi consensus study

**DOI:** 10.1007/s11701-026-04102-0

**Published:** 2026-10-26

**Authors:** George Ryan, Taner Shakir, Nader Francis

**Affiliations:** 1 Division of Surgical and Interventional Sciences, University College London, London, UK; 2 Griffin Institute, Northwick Park Hospital, Harrow, UK

**Keywords:** Robotic surgery, Emergency undocking, Delphi consensus, Patient safety, Conversion, Operating theatre teams

## Abstract

Emergency undocking in robotic surgery is an uncommon but high-stakes event. Published protocols are predominantly single-institution and specialty-specific, and no multidisciplinary cross-specialty consensus exists. This study aimed to establish expert consensus on the definitions and procedural steps of safe emergency undocking. A four-round modified Delphi study was conducted with an international panel of robotic theatre team members. Round 1 used open-text questions to generate themes; Rounds 2 to 4 presented binary statements (Yes / No / Unable to rate), with a pre-specified threshold of 80% agreement and Unable to rate responses excluded from the denominator. Items below threshold, or reaching threshold with substantive comments, were revised and re-presented. Fifty-two experts from 10 countries consented and completed Round 1. The panel comprised lead or experienced console surgeons (53.8%), first assistants (30.8%), surgical care practitioners (9.6%), anaesthetists (3.8%), and one industry representative (1.9%). Response rates were 80.8%, 80.8%, and 76.9% for Rounds 2 to 4 respectively. Thirty-seven statements reached consensus, comprising two definitions, six common pathway items, 18 controlled undocking items, and 11 uncontrolled undocking items. Of the 37 statements, 25 were classified as platform-agnostic and 12 as platform-dependent. Three role-allocation items were deferred to a planned subsequent round. This Delphi process produced 37 consensus statements defining controlled and uncontrolled emergency undocking and the steps for each. The panel was predominantly United Kingdom-based and surgical, and included no scrub or circulating practitioners. The resulting framework requires simulation and clinical validation before implementation.

**Supplementary Information:**

The online version contains supplementary material available at https://doi.org/10.1007/s11701-026-04102-0.

## Introduction

Robotic-assisted surgery has expanded rapidly over the past two decades and is now established across a broad range of surgical specialties [1, 6]. Among the intraoperative risks introduced by the robotic interface, the need for emergency undocking, defined as rapid disengagement of the robotic system from the patient during an intraoperative crisis, is a low-frequency, high-stakes event for which no widely adopted standardised protocol exists [1, 5].

Emergency undocking may be required for uncontrolled haemorrhage, cardiorespiratory arrest, airway emergency or critical equipment failure, and reported conversion rates in robotic surgery are approximately 1% [1]. The robotic system interposes mechanical barriers between surgeon and patient, and the spatial relationship between patient cart, arms, instruments and operative field creates complexity not present in laparoscopic conversion. Errors in sequence, such as moving the cart before arms are disengaged or removing the arm controlling haemorrhage before alternative control is established, carry the potential for catastrophic harm [2, 5].

Existing published protocols are predominantly single-institution and specialty-specific [1, 3–5], and simulation studies show that structured team training measurably reduces missed critical steps during emergency conversion [2]. A recent modified Delphi study established consensus for emergency conversion in robotic thoracic surgery among 21 thoracic surgeons [10]; that study was specialty-specific and surgeon-only, leaving open whether a cross-specialty panel would produce a comparable protocol. Existing protocols also largely address mechanical steps in isolation and do not capture the human factors, leadership and communication elements central to safe team performance in a crisis [2, 11, 12].

This study used a modified Delphi methodology with an international panel drawn from across surgical specialties, comprising console surgeons, first assistants, surgical care practitioners, anaesthetists and one industry representative, to establish consensus on the definitions of controlled and uncontrolled emergency undocking and on the procedural steps required for each pathway.

## Methods

### Study design

A four-round modified Delphi study was prospectively designed and is reported in accordance with the ACcurate COnsensus Reporting Document (ACCORD) guideline [7], with reference to the Guidance on Conducting and REporting DElphi Studies (CREDES) recommendations [8]. A completed ACCORD checklist is provided as Supplementary Material. Round 1 was qualitative, using open-text questions to generate candidate statements; Rounds 2 to 4 were quantitative consensus rounds.

### Panel recruitment and consent

Experts were identified through published literature on robotic surgical complications and emergency protocols, peer nomination, and international robotic surgical society networks. Eligibility was defined by current involvement in robotic operating as a member of the theatre team. A minimum case volume was not applied and direct prior experience of an emergency undocking event was not required. Emergency undocking is sufficiently infrequent that a panel restricted to those with direct experience would have been small and drawn disproportionately from high-volume centres, so we prioritised multinational breadth and role familiarity over depth of direct experience. Expertise in this panel is therefore constructed from current role-specific familiarity with robotic operating, procedural volume, and direct event experience where present. We do not contend that robotic experience alone is equivalent to expertise in emergency undocking, and the implications of this, together with the professional groups absent from the panel, are considered in the Limitations. The specific robotic platform used by each participant was not recorded; participants responded about generic team process and were not asked about system-specific manipulation, which limits the inferences that can be drawn about platform-specific steps. Electronic informed consent was obtained from all participants prior to Round 1 by means of a mandatory declaration confirming willingness to participate and understanding that anonymised aggregate responses would be published.

### Round 1: Qualitative theme generation

Round 1 collected demographic and experiential information, including professional role, country, unit-level protocol status, robotic case volume, personal experience of emergency undocking and preferred team leadership. Four open-text questions elicited definitions of controlled and uncontrolled emergency undocking and the step-by-step procedural actions required for each pathway, including the roles responsible. Free-text responses were analysed to identify candidate themes for conversion into votable statements. The thematic arrangement was performed with the assistance of a large language model (Claude, Anthropic), and each output was then checked by the research team against the source responses to confirm that no theme had been introduced that was not present in the source, merged in a way that lost a distinct position, or omitted. Candidate statements were drafted by the research team from the verified themes. Independent duplicate coding by two investigators working separately was not undertaken and no inter-rater reliability statistic was computed; this is addressed in the Limitations. The four open-text questions are reproduced in Supplementary Table S1.

### Rounds 2 to 4: Consensus rounds

Statements generated from Round 1 were presented as binary items (Yes / No / Unable to rate / Outside my expertise). The consensus threshold was pre-specified at 80% Yes. This was selected as conservative relative to published practice; a systematic review of Delphi studies found percentage agreement to be the most common operationalisation of consensus, with a median threshold of 75% [9]. Items reaching threshold with no substantive comment-derived wording concerns were closed and not re-presented. Items falling below threshold, and items reaching threshold but for which comments identified wording ambiguity or a clinical gap, were revised and re-presented, with explanatory notes in the survey instrument detailing the rationale for change. Free-text comment fields were mandatory for all No responses and optional otherwise. Responses were collected individually and no group discussion took place at any stage; panellists were anonymous to one another throughout, and individual votes and comments are not attributed in this report. The survey instruments were not formally piloted. Comment analysis was conducted iteratively by the research team between rounds.

Round 2 addressed 29 statements covering definitions, the common pathway and the two sequences. Round 3 addressed 17 revised or new statements arising from Round 2 comment analysis. Round 4 addressed three statements on management of the robotic arm controlling active haemorrhage, an area incompletely resolved in Rounds 2 and 3; three rounds were initially anticipated and a fourth was added to resolve it. Three items on allocation of tasks between bedside assistant and scrub practitioner were deferred to a separate planned round and are not reported here.

### Statistical analysis

Percentage agreement was calculated for each binary item. Responses of Unable to rate and Outside my expertise were excluded from the denominator. They were not counted as disagreement, since a participant declining to rate an item outside their role should not be treated as voting against it. The denominator therefore varies between statements within a round, and the statement-specific denominator is reported for every item in Table 4. Descriptive statistics were used throughout. No formal imputation was performed for missing responses.

**Fig. 1:**
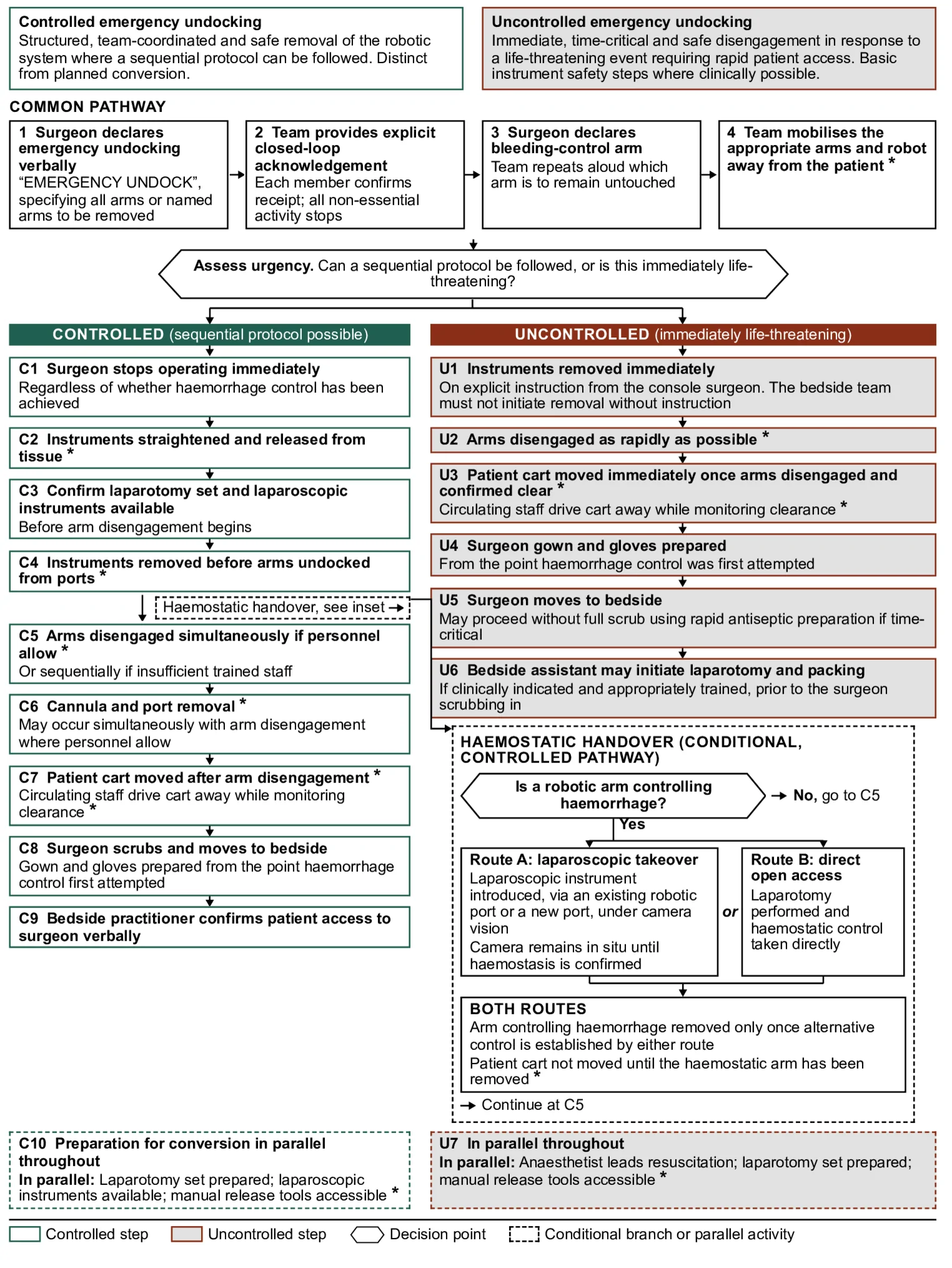
Operational algorithm for emergency undocking in robotic surgery, synthesised from the 37 consensus statements. The content of each step corresponds to a statement that achieved consensus (Table 4). The ordering and branching of steps represent an operational synthesis performed by the research team and were not submitted to the Delphi panel; the sequence has therefore not undergone consensus assessment. Steps marked with an asterisk are platform-dependent (Table 4) and require verification against the manufacturer’s emergency-undocking procedure for the system in local use

Following Round 4 the research team arranged the agreed statements into an operational algorithm (Fig. 1). The sequence was not submitted to the panel and did not undergo consensus assessment. Consensus reported in this study is consensus at the level of individual statements only.

### Use of artificial intelligence

A large language model (Claude, Anthropic) was used in three ways: descriptive statistical computation, which was checked by recalculation against the raw response files; thematic arrangement of Round 1 free-text responses, which was verified by the research team against the source responses as described above; and drafting of survey items and manuscript text, which was reviewed and revised by the named authors. All methodological and clinical decisions and all conclusions are those of the authors, who take full responsibility for the work. The tool does not meet authorship criteria and is not listed as an author.

## Results

### Panel characteristics

Fifty-two experts provided consent and completed Round 1 (100% response rate). Panel characteristics are presented in Table 1 and geographical distribution in Table 2. The panel comprised lead or console surgeons (53.8%), first assistants (30.8%), surgical care practitioners (9.6%), anaesthetists (3.8%) and one industry representative (1.9%), drawn from 10 countries with the United Kingdom providing the largest contingent (55.8%). Robotic case volume was recorded in categories without a specified time period, so these figures indicate the order of magnitude of robotic exposure, with no time period attached: of the 51 participants for whom a category was available, 42 (82.4%) reported at least 50 robotic cases, 30 (58.8%) more than 100 and 18 (35.3%) more than 500. Thirty-five participants (67.3%) had personally experienced at least one emergency undocking event, including 17 (32.7%) who had experienced two or more, with at least 68 events reported in aggregate. Twenty-eight (53.8%) worked in units with a formal emergency-undocking protocol, and the lead or console surgeon was the preferred team leader for 80.8% of respondents.

**Table 1:** Panel characteristics

Characteristic	*n*	%
***Primary role (*** *n* *** = 52)***		
Lead / console surgeon	28	53.8
First assistant	16	30.8
Surgical care practitioner	5	9.6
Anaesthetist	2	3.8
Industry representative	1	1.9
***Robotic case volume (*** *n* *** = 51)***		
Fewer than 50	9	17.6
50–100	12	23.5
101–200	5	9.8
201–500	7	13.7
More than 500	18	35.3
***Personal experience of emergency undocking (*** *n* *** = 52)***		
No previous events	17	32.7
1 event	18	34.6
2 events	8	15.4
3 events	4	7.7
4 events	3	5.8
5 or more events	2	3.8
***Formal emergency undocking protocol in unit (*** *n* *** = 52)***		
Yes	28	53.8
No	17	32.7
Not known	7	13.5
***Preferred team leader during emergency undocking (*** *n* *** = 52)***		
Lead / console surgeon	42	80.8
First assistant	5	9.6
Anaesthetist	1	1.9
Scenario-dependent or shared	4	7.7

**Table 2:** Geographical distribution of panel members

Country	*n*	%
United Kingdom	29	55.8
Italy	6	11.5
India	4	7.7
United States	4	7.7
Belgium	2	3.8
Germany	2	3.8
Spain	2	3.8
Jordan	1	1.9
Malaysia	1	1.9
Turkey	1	1.9
**Total**	**52**	**100.0**

### Round progression

Round 1 was open from 28 November 2025 to 29 March 2026, Round 2 from 1 to 15 May 2026, Round 3 from 10 June to 1 July 2026, and Round 4 from 25 June to 16 July 2026. Response rates were 52/52 (100%), 42/52 (80.8%), 42/52 (80.8%) and 40/52 (76.9%) respectively. Round structure and outcomes are summarised in Table 3.

**Table 3:** Delphi round structure, response rates, and outcomes

Round	Respondents	Format	Items	Outcome
1	52/52 (100%)	Open-text qualitative	Demographics; free-text definitions; procedural steps; leadership	Thematic analysis generated statements for Round 2
2	42/52 (80.8%)	Binary consensus	29 statements	20 closed; 6 revised; 3 deferred
3	42/52 (80.8%)	Binary consensus	17 statements	14 closed; 3 revised
4	40/52 (76.9%)	Binary consensus	3 statements	3 closed; protocol complete

### Consensus outcomes

Thirty-seven statements reached consensus across Rounds 2 to 4 and are presented in Table 4. These comprise two definitions, six common pathway items applicable to all emergency undocking events, 18 items specific to controlled undocking, and 11 items specific to uncontrolled undocking. Agreement ranged from 82.9% to 100%, with 27 of 37 items (73.0%) reaching 95% agreement or above, and nine (24.3%) reaching unanimity.

**Table 4:** Consensus statements by pathway. Round denotes the round in which consensus was reached. n/N denotes Yes votes over valid responses, with Unable to rate excluded. Platform denotes whether the statement applies irrespective of robotic system architecture (Agnostic) or whether its execution, feasibility or timing depends on it (Dependent); the basis for each classification is given in Supplementary Table S2.

Consensus statement	Round	*n*/*N*	% Yes	Platform
**DEFINITIONS**				
Controlled emergency undocking: a structured, team-coordinated, and safe removal of the robotic system in response to an intraoperative emergency where a sequential protocol can be followed; distinct from planned conversion	3	42/42	**100.0**	Agnostic
Uncontrolled emergency undocking: an immediate, time-critical, and safe disengagement of the robotic system in response to a life-threatening intraoperative event requiring rapid patient access; basic instrument safety steps should be followed where clinically possible	3	39/42	**92.9**	Agnostic
**COMMON PATHWAY**				
Emergency undocking should be explicitly declared verbally by the surgeon	2	42/42	**100.0**	Agnostic
The declaration should specify whether all arms or named arms are to be removed	3	38/41	**92.7**	Agnostic
Team members should provide explicit closed-loop acknowledgement of the declaration	3	41/42	**97.6**	Agnostic
Team members should acknowledge by stopping all non-essential activity immediately	2	42/42	**100.0**	Agnostic
The team should repeat aloud which arm is to remain untouched	2	37/42	**88.1**	Agnostic
The team should mobilise the appropriate arms and robot away from the patient	2	42/42	**100.0**	**Dependent**
**CONTROLLED UNDOCKING**				
Once the decision to undock is made, the console surgeon should stop operating immediately, regardless of whether haemorrhage control has been achieved	3	34/41	**82.9**	Agnostic
The surgeon should declare that a specific arm is controlling bleeding and should remain in place	2	41/42	**97.6**	Agnostic
Instruments should be straightened and released from tissue before removal	2	39/41	**95.1**	**Dependent**
A laparoscopic instrument should be available for haemorrhage control	2	40/41	**97.6**	Agnostic
Manual instrument release tools should be immediately accessible	2	40/40	**100.0**	**Dependent**
Instruments should be removed before arms are undocked from ports	2	40/42	**95.2**	**Dependent**
Verbal confirmation that the laparotomy set and laparoscopic instruments are available should be obtained before arm disengagement begins	3	38/42	**90.5**	Agnostic
The arm controlling haemorrhage should not be removed until an alternative means of haemostatic control has been established, whether via laparoscopic instrument or direct open access	4	40/40	**100.0**	Agnostic
Where a laparoscopic instrument takes over haemostatic control, the camera should remain in situ until haemostasis is confirmed	4	39/40	**97.5**	Agnostic
The patient cart should not be moved until the arm controlling haemorrhage has been removed	4	36/38	**94.7**	**Dependent**
Robotic arms may be disengaged simultaneously rather than sequentially, provided sufficient trained personnel are available	3	39/40	**97.5**	**Dependent**
Cannula and port removal may occur simultaneously with arm disengagement where personnel allow	3	38/42	**90.5**	**Dependent**
The patient cart should be moved away only after arm disengagement	2	40/42	**95.2**	**Dependent**
Circulating staff should remove the patient cart while monitoring clearance	2	40/41	**97.6**	**Dependent**
The surgeon should scrub and move to the bedside to assist	2	40/42	**95.2**	Agnostic
A clear verbal confirmation of patient access should be given	2	40/42	**95.2**	Agnostic
Confirmation of patient access should be directed from the bedside practitioner to the console surgeon	3	37/41	**90.2**	Agnostic
Preparation for conversion should occur in parallel	2	41/42	**97.6**	Agnostic
**UNCONTROLLED UNDOCKING**				
If control of bleeding is not achievable or cardiorespiratory compromise is present, instruments should be removed immediately	2	42/42	**100.0**	Agnostic
The bedside team should not initiate instrument removal without explicit instruction from the console surgeon	3	41/42	**97.6**	Agnostic
Arms should be disengaged as rapidly as possible	2	41/42	**97.6**	**Dependent**
The patient cart should be moved away immediately once arms are disengaged and confirmed clear	3	41/42	**97.6**	**Dependent**
Circulating staff should drive the robot cart away while monitoring clearance	2	41/42	**97.6**	**Dependent**
The anaesthetist should lead resuscitation, communicate urgency, and prepare for CPR or defibrillation as required	2	40/42	**95.2**	Agnostic
The laparotomy set should be prepared in parallel	2	41/42	**97.6**	Agnostic
Surgeon gown and gloves should be prepared	2	41/41	**100.0**	Agnostic
Gown and gloves should be prepared from the point at which haemorrhage control is first attempted	3	39/42	**92.9**	Agnostic
The bedside assistant may initiate laparotomy and packing prior to the console surgeon scrubbing in, if clinically indicated and appropriately trained	3	42/42	**100.0**	Agnostic
Where immediate open access is required, the surgeon may proceed without full scrub using rapid antiseptic preparation	3	37/41	**90.2**	Agnostic

Both definitions were revised during the study and reached consensus in Round 3 at 100% and 92.9% respectively. Seven procedural statements required revision and re-presentation across rounds, covering the trigger for ceasing to operate, cannula and port removal timing, sequential versus simultaneous arm disengagement, patient cart movement timing, and management of the arm controlling haemorrhage. One statement, concerning insertion of a laparoscopic port before removal of the haemostatic arm, fell below threshold at 66.7% in Round 3 and was reformulated for Round 4. Three role-allocation items reached the numerical threshold in Round 2 but were deferred following comment-derived objections to fixed spatial role assignment. 

Each agreed statement was classified according to whether its execution depends on robotic system architecture (Table 4). Of the 37 statements, 25 (67.6%) address declaration, closed-loop communication, decision-making, role behaviour, equipment preparation or resuscitation and apply irrespective of architecture. The remaining 12 (32.4%) depend on the number and independence of patient carts, the arm-detachment mechanism, the instrument-release mechanism, or the coupling between arm and cannula. The classification was performed by the research team and was not submitted to the panel.

### The consensus-derived operational algorithm

The agreed statements were synthesised by the research team into an operational algorithm (Fig. 1) comprising a common pathway that bifurcates into controlled and uncontrolled sequences following an assessment of urgency. The ordering of steps within the algorithm was not submitted to the panel and carries no consensus.

The common pathway comprises verbal declaration of emergency undocking by the surgeon, specifying whether all arms or named arms are to be removed; closed-loop acknowledgement by team members with cessation of non-essential activity; and declaration of the bleeding-control arm, repeated aloud by the team.

In the controlled pathway the console surgeon stops operating once the decision to undock is made, regardless of whether haemorrhage control has been achieved. Instruments are straightened, released from tissue and removed before arms are undocked. Where a robotic arm is controlling haemorrhage it is retained until alternative control is established by either laparoscopic instrument or direct open access, with the camera remaining in situ where the laparoscopic route is used and the patient cart not moved until that arm has been removed. Arms may then be disengaged simultaneously where sufficient trained personnel are available, with cannula and port removal concurrent where staffing allows; the cart is withdrawn by circulating staff monitoring clearance and patient access is confirmed to the console surgeon, with conversion preparation proceeding in parallel throughout. Agreement figures for each statement are given in Table 4.

In the uncontrolled pathway instruments are removed immediately where bleeding control is not achievable or cardiorespiratory compromise is present, on explicit instruction from the console surgeon; arms are disengaged as rapidly as possible and the cart moved once arms are confirmed clear. The surgeon may proceed to the operative field without full scrub using rapid antiseptic preparation, and the bedside assistant may initiate laparotomy and packing prior to the surgeon scrubbing in if clinically indicated and appropriately trained. Anaesthetist-led resuscitation, laparotomy set preparation, and preparation of gown and gloves from the point haemorrhage control is first attempted proceed in parallel.

## Discussion

This study presents an international consensus framework for emergency undocking in robotic surgery. Across four Delphi rounds with a 52-member panel, 37 statements reached consensus: 25 apply irrespective of robotic system architecture and 12 depend on it, requiring verification against manufacturer-specific procedures before local use.

### Structure of the protocol

The framework distinguishes a common pathway from pathway-specific sequences, reflecting that steps preceding haemorrhage management are largely shared while the urgency of the precipitating event determines subsequent priorities.

The controlled and uncontrolled distinction was itself contested. A minority argued that the terms should describe the precipitating event. On their view the undocking process should remain systematic regardless of urgency, and one respondent noted that the term controlled emergency implies that an uncontrolled emergency is acceptable. This was accommodated by revising the uncontrolled definition. The original wording, that standard sequencing may not be possible, was considered by several respondents to license unsafe improvisation, and was replaced with a statement that basic instrument safety steps should be followed where clinically possible. The criterion of physiological stability was removed from the controlled definition, since a patient may remain unstable from a haemorrhage that has since been controlled. The word safe was added to both definitions at the panel’s request.

### Haemostatic handover

Management of the arm controlling active haemorrhage was the most technically complex area of the study and required three rounds to resolve. The initial formulation presupposed insertion of a new laparoscopic port before the haemostatic arm could be removed. This was rejected at 66.7%, respondents noting that existing robotic ports accommodate laparoscopic instruments directly once an arm is undocked, making an additional port an unnecessary step.

A second and more substantial objection was that the laparoscopic route may not occur at all. Several respondents indicated that in practice the haemostatic arm is retained until direct open access is achieved via laparotomy, one noting that the laparoscopic scenario is rare. The reformulated statement, that the haemostatic arm should not be removed until alternative control is established by either laparoscopic or open means, reached unanimous agreement in Round 4. Protocols describing only the laparoscopic route give no guidance for what respondents indicated is the more common scenario, and this two-route structure is a contribution relative to existing published protocols.

Our findings converge with the thoracic-specific Delphi consensus of Reddington and colleagues, which independently identified initial tamponade with robotic instruments, conversion via a partial undocking protocol, and uninterrupted visualisation as essential principles [10]. That these emerged from a separate panel, specialty and methodology provides convergent validity for the haemostatic arm and camera retention statements agreed here. The present study extends that work across surgical specialties and by addressing the uncontrolled pathway.

### Platform dependency

Integrated systems mount multiple arms on a single boom and cart, so withdrawal of the cart withdraws all arms together, whereas modular systems mount each arm on an independent cart that can be withdrawn individually. Twelve statements are affected. The statement that the patient cart should not be moved until the arm maintaining haemostatic control has been removed is a necessary safety rule in an integrated system; in a modular system that arm may occupy an independent cart while others are withdrawn, so the statement as worded is more restrictive than the underlying principle requires.

We have not reworded the voted statements, since that wording is what agreement was obtained on, but we identify the affected statements so they can be adapted locally with the intent preserved. Local implementation of any platform-dependent statement requires verification against the manufacturer’s emergency-undocking procedure, and where the two conflict the manufacturer’s instruction should take precedence. The observation by two Round 4 participants that the cart drive mechanism is disabled on some platforms while any arm remains docked is an example of a hardware interlock already enforcing what one statement asks of the team.

Several new platforms are entering clinical use, some of which may incorporate emergency-release features that render specific steps unnecessary. The classification in Table 4 is intended to make the framework extensible: platform-agnostic statements describe team behaviour and decision logic and should remain applicable, while platform-dependent statements are those a manufacturer’s design may alter or eliminate, and require re-verification when a centre adopts a new system.

### Sequential versus simultaneous disengagement

The statement that robotic arms should be disengaged sequentially produced the lowest agreement among items subsequently revised and closed, at 70.7%. Comments rejected the requirement to disengage sequentially while accepting the practice itself, noting that with sufficient trained personnel simultaneous disengagement is faster and equally safe, and that the optimal order depends on which instrument can be removed first and which arm is controlling bleeding. The revised statement, permitting simultaneous disengagement where sufficient trained personnel are available, reached 97.5%, and argues for protocol design that accommodates variation in staffing. A universal order should not be imposed.

### Ambiguity in the operative cessation trigger

The Round 2 statement that the console surgeon should stop operating immediately after obtaining control reached 85.0% but was identified through comments as ambiguous and potentially clinically incorrect. Respondents read obtaining control variously as haemorrhage, instrument or situational control, and the wording implied that the surgeon should continue operating until control is achieved, which is inappropriate where console haemorrhage control is not achievable. The revised statement, that the surgeon should stop once the decision to undock has been made regardless of whether haemorrhage control has been achieved, reached 82.9%.

### Future directions

It could reasonably be argued that simulation testing should have preceded publication. We report the consensus first because a simulation study requires an agreed statement set to test, agreed definitions to distinguish scenarios, and agreed critical-error definitions to score performance, none of which previously existed other than institution-specific.

The framework has not been tested in use. We propose a two-stage validation programme, specified in Table 5: a simulation-based randomised comparison of intact theatre teams across three emergency undocking scenarios, stratified by platform architecture, with time from declaration to confirmed patient access as the primary outcome; followed by prospective multicentre capture of real events, since simulation can establish feasibility, speed and error frequency but not clinical benefit. Recruiting intact teams would also represent the scrub and circulating roles absent from this panel where their actions are tested.

**Table 5:** Proposed two-stage validation programme. Neither stage has been undertaken

Stage	Domain	Specification
Stage 1	Design	Multicentre randomised comparison. Intact theatre teams allocated to receive the framework with structured briefing and walkthrough, or to manage scenarios according to current local practice. Team is the unit of randomisation and analysis.
Stage 1	Stratification	By platform architecture: integrated multi-arm single-cart versus modular independent-arm-cart systems, so that platform-dependent statements are tested under both.
Stage 1	Participants	Intact teams: console surgeon, bedside assistant, scrub practitioner, circulating practitioner and anaesthetist, with an operating department practitioner where this reflects local establishment.
Stage 1	Setting	Robotic simulation suite; draped high-fidelity mannequin docked to a clinically representative platform.
Stage 1	Scenario 1	Controlled undocking: progressive bleeding where haemostatic control is achievable and must be maintained by one arm.
Stage 1	Scenario 2	Uncontrolled undocking: intraoperative cardiac arrest requiring immediate chest access and CPR.
Stage 1	Scenario 3	Uncontrolled undocking: catastrophic vascular injury where control is not achievable.
Stage 1	Primary outcome	Time from verbal declaration of emergency undocking to confirmed patient access, scored from theatre video by two assessors.
Stage 1	Secondary outcomes	Time to complete instrument removal; time to cart clearance; critical error frequency; non-technical skills using a validated instrument; cognitive load (NASA Task Load Index); usability by structured post-scenario questionnaire.
Stage 1	Critical errors	Movement of a patient cart carrying the arm maintaining haemostatic control; withdrawal of an instrument still engaged in tissue; removal of the camera before haemostasis is confirmed by an alternative means; arm disengagement initiated without closed-loop acknowledgement of the declaration.
Stage 1	Blinding	Assessors blinded to allocation so far as the recordings allow. Complete blinding is not achievable in this design; the extent to which allocation was apparent will be reported.
Stage 1	Sample size	Approximately 28 teams per arm, assuming a mean time to patient access of 180 s, standard deviation 60 s, detection of a 45-second difference, 80% power and two-sided alpha of 0.05.
Stage 1	Debrief	Video-assisted debrief with structured think-aloud after each scenario, to establish why individual steps succeed or fail.
Stage 2	Design	Prospective multicentre registry of consecutive emergency undocking events, using a one-page standardised proforma completed immediately after the event.
Stage 2	Data captured	Time to patient access; deviations from the framework and the reason for each; equipment or platform-specific problems; patient outcomes including estimated blood loss, transfusion, conversion, critical care admission and 30-day morbidity and mortality.
Stage 2	Expected yield	A 20-centre network performing approximately 400 robotic procedures per centre per year accrues approximately 16,000 procedures over 24 months. The incidence of emergency undocking is not well characterised; this would yield of the order of 16 events at 0.1%, 80 at 0.5% and 160 at 1%.
Stage 2	Interpretation	Sufficient to describe deviation patterns and equipment failures. Demonstration of a difference in patient outcome would require international collaboration using common data definitions.

### Limitations

The panel’s composition constrains generalisability. Twenty-nine of 52 participants (55.8%) were based in the United Kingdom, so the framework reflects UK theatre staffing and governance more closely than other systems. The panel was predominantly surgical: 44 (84.6%) were console surgeons or first assistants, 5 (9.6%) surgical care practitioners, 2 (3.8%) anaesthetists and 1 (1.9%) an industry representative. No participant identified their primary role as scrub practitioner, circulating practitioner or operating department practitioner, yet several statements specify actions performed by those groups, including withdrawal of the patient cart by circulating staff. Similarly, the statement that the anaesthetist should lead resuscitation achieved 95.2% agreement, but that represents what a surgical majority expects of anaesthetists. With two anaesthetists on the panel it is not an anaesthetic consensus. Statements of this kind require endorsement by the relevant professions before adoption as role specifications.

Response rates declined from 100% in Round 1 to 76.9% in Round 4, introducing the possibility of responder bias in later rounds.

The robotic platform used by each participant was not recorded, so we cannot determine whether agreement on the 12 platform-dependent statements was uniform across architectures. Given the predominantly United Kingdom and European composition of the panel, most respondents’ experience probably relates to integrated multi-arm platforms with a single patient cart, which would bias those statements toward that architecture. Two Round 4 respondents noted that the cart drive mechanism is disabled while any arm remains docked on some platforms, which would mean the corresponding statement describes a hardware interlock the system already enforces. Future consensus work should record platform at enrolment and report agreement stratified by architecture.

Theatre staffing was a recurring theme in free-text responses and is reflected in the conditional wording of two statements, which permit parallel action only where sufficient trained personnel are available. The pathway a team can execute is therefore contingent on its skill mix and staffing at the time, and will differ between a daytime elective list and an out-of-hours emergency.

Consensus was obtained on individual statements and not on their sequence. In emergency management sequence is a determinant of outcome: the order in which instruments are removed, arms disengaged, the cart withdrawn and access confirmed affects both time to access and the risk of injury during undocking. Figure 1 is the research team’s synthesis into a workable order, and alternative orderings consistent with the same 37 statements are possible. A dedicated sequencing round is planned and should precede adoption of the algorithm as a formal protocol.

Participants’ direct experience varied considerably and approximately one third had never been present at an emergency undocking event. A panel restricted to those with direct experience would have been small and drawn disproportionately from high-volume centres. Agreement therefore reflects expert judgement about what should occur. No observational evidence supports it. Because Round 1 responses were anonymous and identifiers optional, with only 28 of 52 respondents supplying an email address, responses could not be linked across rounds and a subgroup analysis of consensus by prior event experience was not possible.

Three statements concerning the allocation of specific responsibilities were deferred to a separate consensus exercise, and this framework does not resolve role allocation. Several statements do carry role content, specifying that the console surgeon declares the emergency and authorises instrument removal, that circulating staff withdraw the cart, that the anaesthetist leads resuscitation, and that the bedside assistant may initiate laparotomy and packing where trained. These are individual agreed statements. They do not amount to an allocated role set: no agreement was sought on who coordinates the sequence, on concurrent activity at each stage, or on how responsibility transfers if a designated individual is unavailable. This compounds the composition limitation above, since the roles whose responsibilities remain unallocated are also those not represented.

This study establishes expert agreement and nothing beyond it. No assessment of undocking time, critical error frequency, usability, team performance or patient outcome has been performed. High agreement is not evidence that acting on a statement is safe, fast or feasible, and steps commanding near-unanimous agreement may prove impracticable under time pressure. The framework should be used to structure local discussion and to design simulation scenarios, not adopted as a clinical protocol in advance of the validation set out in Table 5.

The thematic arrangement of Round 1 free-text responses was AI-assisted, with each output verified by the research team against the source responses to detect introduced, merged or omitted themes. This is a weaker safeguard than independent duplicate coding with formal assessment of agreement, which was not performed. A theme erroneously introduced could only enter the framework by achieving 80% agreement in a panel vote. Omission is the more serious residual risk: a theme missed at the arrangement stage would never have reached the panel, and its absence is not detectable from the results reported here.

### Conclusion

This four-round Delphi process produced 37 consensus statements covering the definitions of controlled and uncontrolled emergency undocking, a shared declaration and acknowledgement pathway, and divergent action sequences thereafter. The research team arranged these into an operational algorithm (Fig. 1). The ordering was not submitted to the panel and carries no consensus. Twenty-five statements apply irrespective of system architecture; 12 depend on it and require verification against the manufacturer’s emergency-undocking procedure. The panel included no scrub or circulating practitioners, and allocation of responsibilities between team members remains unresolved. This work is a starting point for standardisation and for training design, not a validated protocol: prospective simulation and subsequent clinical capture are required before widespread implementation.

## Supplementary Information

Below is the link to the electronic supplementary material.

Supplementary Material 1

Supplementary Material 2

## Data Availability

The datasets generated and analysed during the current study are not publicly available in order to protect the confidentiality of individual panellist responses, but anonymised round-level and item-level data are available from the corresponding author on reasonable request.

## Declarations

### Study registration

This study was not prospectively registered. The consensus threshold and analytic approach were defined a priori and applied without modification, and all rounds and item-level results are reported in full.

### Endorsement

No organisation endorsed or sponsored this consensus exercise.
