# Supplementary material for: Emergency undocking in robotic surgery: an international Delphi consensus study
Source: J Robot Surg. 2026 Oct 26;20(1):1135. doi: 10.1007/s11701-026-04102-0 (PMC13696567; doi:10.1007/s11701-026-04102-0)
Supplement: Supplementary file 1 [file 11701_2026_4102_MOESM1_ESM.docx]

**ACCORD Checklist**

*Emergency Undocking in Robotic Surgery: An International Multidisciplinary Delphi Consensus Protocol*

Ryan G, Shakir T, Francis N

Item wording reproduced from Table 4 of Gattrell WT, Logullo P, van Zuuren EJ, et al. ACCORD (ACcurate COnsensus Reporting Document): a reporting guideline for consensus methods in biomedicine developed via a modified Delphi. PLoS Med. 2024;21(1):e1004326.

*All 35 items are addressed. Items marked not applicable are stated as such with justification. Page numbers refer to manuscript version 3 and should be confirmed against the final paginated submission.*

| **Item** | **Section** | **Item wording** | **Reported in** | **Page** |
| --- | --- | --- | --- | --- |
| **TITLE** | | | | |
| **T1** | Title | Identify the article as reporting a consensus exercise and state the consensus methods used in the title. | Title identifies the article as an international Delphi consensus study and names the consensus method used | 1 |
| **INTRODUCTION** | | | | |
| **I1** | Introduction | Explain why a consensus exercise was chosen over other approaches. | Introduction, paragraphs 2-3: emergency undocking is a low-frequency event for which trial evidence is not feasible; existing protocols are single-institution and specialty-specific. | 4-5 |
| **I2** | Introduction | State the aim of the consensus exercise, including its intended audience and geographical scope (national, regional, global). | Introduction, final paragraph: aim stated; international scope; intended for multidisciplinary robotic theatre teams across surgical specialties. | 5 |
| **I3** | Introduction | If the consensus exercise is an update of an existing document, state why an update is needed, and provide the citation for the original document. | Not applicable. This is not an update of an existing document. | - |
| **METHODS** | | | | |
| **M1** | Registration | If the study or study protocol was prospectively registered, state the registration platform and provide a link. If the exercise was not registered, this should be stated. | Declarations, Study registration: stated that the study was not prospectively registered; threshold and analytic approach defined a priori | 30 |
| **M2** | Selection of SC and/or panellists | Describe the role(s) and areas of expertise or experience of those directing the consensus exercise. | Methods, Rounds 2 to 4; Author contributions. The research team comprised two general surgeons and a consultant colorectal surgeon; none voted. | 7-8, 30 |
| **M3** | Selection of SC and/or panellists | Explain the criteria for panellist inclusion and the rationale for panellist numbers. State who was responsible for panellist selection. | Methods, Panel recruitment and consent: inclusion criteria stated; panel size guided by multidisciplinary and international representation; selection by the research team. | 6-7 |
| **M4** | Selection of SC and/or panellists | Describe the recruitment process (how panellists were invited to participate). | Methods, Panel recruitment and consent: identified through published literature, peer nomination and robotic surgical society networks; invited by email. | 6-7 |
| **M5** | Selection of SC and/or panellists | Describe the role of any members of the public, patients, or carers in the different steps of the study. | Not applicable. The consensus addressed intraoperative technical and team procedure; no patients, carers or members of the public participated. | - |
| **M6** | Preparatory research | Describe how information was obtained prior to generating items or other materials used during the consensus exercise. | Methods, Round 1: candidate statements generated from thematic analysis of panellist free-text responses rather than from a literature-derived list. | 7 |
| **M7** | Preparatory research | Describe any systematic literature search in detail, including the search strategy and dates of search or the citation if published already. | Not applicable. No systematic literature search was conducted; items were generated de novo from Round 1 panellist responses. | - |
| **M8** | Preparatory research | Describe how any existing scientific evidence was summarized and if this evidence was provided to the panellists. | No evidence summary was provided to panellists. Items were derived solely from panellist experience elicited in Round 1. | 7 |
| **M9** | Assessing consensus | Describe the methods used and steps taken to gather panellist input and reach consensus (for example, Delphi, RAND/UCLA, nominal group technique). | Methods, Study design and Rounds 2 to 4: four-round modified Delphi; modification comprised a qualitative generative first round and binary rather than Likert voting. | 6-8 |
| **M10** | Assessing consensus | Describe how each question or statement was presented and the response options. State whether panellists were able to or required to explain their responses, and whether they could propose new items. | Methods, Rounds 2 to 4: binary items (Yes / No / Unable to rate / Outside my expertise); free-text mandatory for all No responses, optional otherwise. Round 1 questions in Supplementary Table S1. | 7-8, 34 |
| **M11** | Assessing consensus | State the objective of each consensus step. | Methods, Rounds 2 to 4, final paragraph: objective of each round stated (Round 2 initial statements, Round 3 revisions, Round 4 haemostatic arm management). | 8 |
| **M12** | Assessing consensus | State the definition of consensus (for example, number, percentage, or categorical rating, such as ‘agree’ or ‘strongly agree’) and explain the rationale for that definition. | Methods, Rounds 2 to 4 and Statistical analysis: 80% Yes pre-specified, with Unable to rate and Outside my expertise excluded from the denominator and the statement-specific denominator reported for every item in Table 4 | 7-9 |
| **M13** | Assessing consensus | State whether items that met the prespecified definition of consensus were included in any subsequent voting rounds. | Methods, Rounds 2 to 4: items reaching threshold without comment-derived concerns were closed and were not re-presented in subsequent rounds; items reaching threshold but with identified wording or clinical concerns were revised and re-presented. | 7-8 |
| **M14** | Assessing consensus | For each step, describe how responses were collected, and whether responses were collected in a group setting or individually. | Methods, Study design and Rounds 2 to 4: all rounds administered electronically; responses were collected individually and no group discussion took place at any stage. | 6-8 |
| **M15** | Assessing consensus | Describe how responses were processed and/or synthesized. | Methods, Round 1 and Use of artificial intelligence: thematic arrangement of free-text responses was AI-assisted (large language model, Claude, Anthropic), with each output checked by the research team against the source responses for introduced, merged or omitted themes; independent duplicate coding by two investigators and inter-rater reliability statistics were not undertaken, and this is stated as a limitation | 7, 9, 26 |
| **M16** | Assessing consensus | Describe any piloting of the study materials and/or survey instruments. | Methods, Rounds 2 to 4: stated that the survey instruments were not formally piloted prior to distribution. | 8 |
| **M17** | Assessing consensus | If applicable, describe how feedback was provided to panellists at the end of each consensus step or meeting. | Methods, Rounds 2 to 4: revised items were accompanied by explanatory notes in the survey instrument detailing the rationale for change. | 7-8 |
| **M18** | Assessing consensus | State whether anonymity was planned in the study design. Explain where and to whom it was applied and what methods were used to guarantee anonymity. | Methods, Rounds 2 to 4: panellists were anonymous to one another throughout; individual votes and comments are not attributed. Limitations notes that Round 1 identifiers were optional, preventing linkage across rounds. | 8, 24 |
| **M19** | Assessing consensus | State if the steering committee was involved in the decisions made by the consensus panel. | Methods, Rounds 2 to 4 and Author contributions: the research team generated and revised statements and conducted comment analysis but did not vote. | 7-8, 30 |
| **M20** | Participation | Describe any incentives used to encourage responses or participation in the consensus process. | Methods, Panel recruitment and consent: no incentives or remuneration were offered; email reminders were issued prior to each round deadline. | 6-7 |
| **M21** | Participation | Describe any adaptations to make the surveys/meetings more accessible. | Methods, Panel recruitment and consent: all rounds were administered in English; no translated or plain-language versions were provided. | 6-7 |
| **RESULTS** | | | | |
| **R1** | Results | State when the consensus exercise was conducted. List the date of initiation and the time taken to complete each consensus step, analysis, and any extensions or delays in the analysis. | Results, Round progression: Round 1 open 28 November 2025 to 29 March 2026; Round 2 1 to 15 May 2026; Round 3 10 June to 1 July 2026; Round 4 25 June to 16 July 2026. | 12 |
| **R2** | Results | Explain any deviations from the study protocol, and why these were necessary. | Methods, Rounds 2 to 4: three rounds were initially anticipated; a fourth was added to resolve management of the arm controlling haemorrhage. | 8 |
| **R3** | Results | For each step, report quantitative (number of panellists, response rate) and qualitative (relevant sociodemographics) data to describe the participating panellists. | Results, Panel characteristics and Round progression; Tables 1, 2 and 3. | 10-12 |
| **R4** | Results | Report the final outcome of the consensus process as qualitative (for example, aggregated themes from comments) and/or quantitative (for example, summary statistics, score means, medians, and/or ranges) data. | Results, Consensus outcomes and Table 4 (quantitative, n/N and percentage for all 37 items); Discussion (aggregated themes from free-text comments). | 12-16 |
| **R5** | Results | List any items or topics that were modified or removed during the consensus process. Include why and when in the process they were modified or removed. | Results, Consensus outcomes: seven procedural statements and both definitions revised, with round identified; one item below threshold at 66.7% reformulated for Round 4; three role-allocation items deferred. | 13 |
| **DISCUSSION** | | | | |
| **D1** | Discussion | Discuss the methodological strengths and limitations of the consensus exercise. | Discussion, Limitations (nine paragraphs): panel composition and professional groups not represented; response rate decline; robotic platform not recorded; theatre staffing; statement-level consensus versus sequence; definition of expertise and inability to link responses across rounds; deferred role allocation; absence of operational validation; AI assistance in thematic analysis | 24-27 |
| **D2** | Discussion | Discuss whether the recommendations are consistent with any preexisting literature and, if not, propose reasons why this process may have arrived at alternative conclusions. | Discussion, Haemostatic handover and Platform dependency: convergence with Reddington et al.; platform-dependent statements identified and requirement for manufacturer verification stated | 20-22 |
| **OTHER INFORMATION** | | | | |
| **O1** | Other information | List any endorsing organizations involved and their role. | Declarations, Endorsement: no organisation endorsed or sponsored this consensus exercise. | 29 |
| **O2** | Other information | State any potential conflicts of interests, including among those directing the consensus study and panellists. Describe how conflicts of interest were managed. | Declarations, Competing interests: author interests declared; panellists were not asked to declare individual conflicts; participation was unremunerated and no commercial sponsor was involved. | 29 |
| **O3** | Other information | State any funding received and the role of the funder. | Declarations, Funding: no specific funding received. | 29 |

*SC, Steering Committee. Items marked not applicable are stated as such with justification, consistent with ACCORD guidance that the checklist is a reporting guideline rather than a methodological mandate.*
