# Supplementary material for: Emergency undocking in robotic surgery: an international Delphi consensus study
Source: J Robot Surg. 2026 Oct 26;20(1):1135. doi: 10.1007/s11701-026-04102-0 (PMC13696567; doi:10.1007/s11701-026-04102-0)
Supplement: Supplementary file 2 [file 11701_2026_4102_MOESM2_ESM.docx]

**Supplementary Table S1**

Open-text questions presented in Round 1.

| **Domain** | **Open-text question** |
| --- | --- |
| Controlled emergency undocking: definition | How would you define a controlled emergency undocking? Please describe this definition in your own words. The study team will use your input to identify areas of consensus and disagreement for refinement in later rounds. |
| Uncontrolled emergency undocking: definition | How would you define an uncontrolled emergency undocking? Please describe this definition in your own words. The study team will use your input to identify areas of consensus and disagreement for refinement in later rounds. |
| Controlled emergency undocking: procedural steps | In your experience, what essential steps should be taken by someone in your role to complete a safe controlled emergency undock? Please describe in chronological order the steps you would take, including the role(s) responsible for each: surgeon, assistant, anaesthetist, operating department practitioner, scrub nurse and circulator. |
| Uncontrolled emergency undocking: procedural steps | In your experience, what essential steps should be taken by someone in your role to complete a safe uncontrolled emergency undock? Please describe in chronological order the steps you would take, including the role(s) responsible for each: surgeon, assistant, anaesthetist, operating department practitioner, scrub nurse and circulator. |

**Supplementary Table S2. Basis for platform classification of the 37 consensus statements. Classification was performed by the research team following completion of the consensus process and was not submitted to the panel.**

| **Section** | **Consensus statement** | **Classification** | **Basis** |
| --- | --- | --- | --- |
| Definitions | Controlled emergency undocking: a structured, team-coordinated, and safe removal of the robotic system in response to an intraoperative emergency where a sequential protocol can be followed; distinct from planned conversion | Agnostic | Definitional; describes the clinical circumstance, not a system manipulation |
| Definitions | Uncontrolled emergency undocking: an immediate, time-critical, and safe disengagement of the robotic system in response to a life-threatening intraoperative event requiring rapid patient access; basic instrument safety steps should be followed where clinically possible | Agnostic | Definitional; the qualifier “where clinically possible” already accommodates system differences |
| Common Pathway | Emergency undocking should be explicitly declared verbally by the surgeon | Agnostic | Communication act |
| Common Pathway | The declaration should specify whether all arms or named arms are to be removed | Agnostic | Communication act; applies to any multi-arm system |
| Common Pathway | Team members should provide explicit closed-loop acknowledgement of the declaration | Agnostic | Communication act |
| Common Pathway | Team members should acknowledge by stopping all non-essential activity immediately | Agnostic | Team behaviour |
| Common Pathway | The team should repeat aloud which arm is to remain untouched | Agnostic | Communication act |
| Common Pathway | The team should mobilise the appropriate arms and robot away from the patient | Dependent | “The robot” is a single cart in integrated systems but multiple independent carts in modular systems; the action becomes per-arm and may require parallel personnel |
| Controlled Undocking | Once the decision to undock is made, the console surgeon should stop operating immediately, regardless of whether haemorrhage control has been achieved | Agnostic | Decision rule |
| Controlled Undocking | The surgeon should declare that a specific arm is controlling bleeding and should remain in place | Agnostic | Communication act |
| Controlled Undocking | Instruments should be straightened and released from tissue before removal | Dependent | Instrument articulation and release mechanisms differ by platform; the manoeuvre required is system-specific |
| Controlled Undocking | A laparoscopic instrument should be available for haemorrhage control | Agnostic | Equipment preparation |
| Controlled Undocking | Manual instrument release tools should be immediately accessible | Dependent | Manual release mechanism, tooling and location are manufacturer-specific; some systems require no separate tool |
| Controlled Undocking | Instruments should be removed before arms are undocked from ports | Dependent | Depends on coupling between arm and cannula and whether instrument withdrawal and arm detachment are separable steps |
| Controlled Undocking | Verbal confirmation that the laparotomy set and laparoscopic instruments are available should be obtained before arm disengagement begins | Agnostic | Communication and equipment check |
| Controlled Undocking | The arm controlling haemorrhage should not be removed until an alternative means of haemostatic control has been established, whether via laparoscopic instrument or direct open access | Agnostic | Safety principle; deliberately worded to permit either route |
| Controlled Undocking | Where a laparoscopic instrument takes over haemostatic control, the camera should remain in situ until haemostasis is confirmed | Agnostic | Safety principle applicable to any system with a separate endoscope arm |
| Controlled Undocking | The patient cart should not be moved until the arm controlling haemorrhage has been removed | Dependent | Presupposes the haemostatic arm is on the cart being moved; in modular systems it may sit on an independent cart that can remain while others are withdrawn |
| Controlled Undocking | Robotic arms may be disengaged simultaneously rather than sequentially, provided sufficient trained personnel are available | Dependent | Feasibility and personnel requirement differ between integrated and modular architectures; the personnel proviso acquires additional force in modular systems |
| Controlled Undocking | Cannula and port removal may occur simultaneously with arm disengagement where personnel allow | Dependent | Depends on cannula mounting and whether arm detachment and cannula removal are mechanically coupled |
| Controlled Undocking | The patient cart should be moved away only after arm disengagement | Dependent | In modular systems this becomes a per-cart instruction, with each cart withdrawn separately |
| Controlled Undocking | Circulating staff should remove the patient cart while monitoring clearance | Dependent | Number of carts to be withdrawn, and therefore staff required, is architecture-dependent |
| Controlled Undocking | The surgeon should scrub and move to the bedside to assist | Agnostic | Role behaviour |
| Controlled Undocking | A clear verbal confirmation of patient access should be given | Agnostic | Communication act |
| Controlled Undocking | Confirmation of patient access should be directed from the bedside practitioner to the console surgeon | Agnostic | Communication act |
| Controlled Undocking | Preparation for conversion should occur in parallel | Agnostic | Team process |
| Uncontrolled Undocking | If control of bleeding is not achievable or cardiorespiratory compromise is present, instruments should be removed immediately | Agnostic | Decision rule |
| Uncontrolled Undocking | The bedside team should not initiate instrument removal without explicit instruction from the console surgeon | Agnostic | Communication and authority rule |
| Uncontrolled Undocking | Arms should be disengaged as rapidly as possible | Dependent | Achievable speed, and whether disengagement is serial or parallel, is determined by arm-detachment mechanism and cart architecture |
| Uncontrolled Undocking | The patient cart should be moved away immediately once arms are disengaged and confirmed clear | Dependent | Single-cart instruction; becomes per-cart in modular systems |
| Uncontrolled Undocking | Circulating staff should drive the robot cart away while monitoring clearance | Dependent | Cart number, steering mechanism and clearance envelope are platform-specific |
| Uncontrolled Undocking | The anaesthetist should lead resuscitation, communicate urgency, and prepare for CPR or defibrillation as required | Agnostic | Role behaviour and resuscitation |
| Uncontrolled Undocking | The laparotomy set should be prepared in parallel | Agnostic | Equipment preparation |
| Uncontrolled Undocking | Surgeon gown and gloves should be prepared | Agnostic | Equipment preparation |
| Uncontrolled Undocking | Gown and gloves should be prepared from the point at which haemorrhage control is first attempted | Agnostic | Timing of preparation; independent of system |
| Uncontrolled Undocking | The bedside assistant may initiate laparotomy and packing prior to the console surgeon scrubbing in, if clinically indicated and appropriately trained | Agnostic | Role behaviour and competence |
| Uncontrolled Undocking | Where immediate open access is required, the surgeon may proceed without full scrub using rapid antiseptic preparation | Agnostic | Asepsis trade-off; independent of system |
